# Hsa-miR-210-3p expression in breast cancer and its putative association with worse outcome in patients treated with Docetaxel

**DOI:** 10.1038/s41598-019-51581-3

**Published:** 2019-10-17

**Authors:** Barbara Pasculli, Raffaela Barbano, Michelina Rendina, Andrea Fontana, Massimiliano Copetti, Tommaso Mazza, Vanna Maria Valori, Maria Morritti, Evaristo Maiello, Paolo Graziano, Roberto Murgo, Vito Michele Fazio, Manel Esteller, Paola Parrella

**Affiliations:** 1 Fondazione IRCCS Casa Sollievo della Sofferenza Laboratorio di Oncologia, San Giovanni Rotondo, FG Italy; 2 Fondazione IRCCS Casa Sollievo della Sofferenza, UO di Biostatistica, San Giovanni Rotondo, FG Italy; 3 Fondazione IRCCS Casa Sollievo della Sofferenza, Bioinformatics Unit, San Giovanni Rotondo, FG Italy; 4 Fondazione IRCCS Casa Sollievo della Sofferenza, UO di Oncologia, San Giovanni Rotondo, FG Italy; 5 Fondazione IRCCS Casa Sollievo della Sofferenza, UO di Anatomia Patologica, San Giovanni Rotondo, FG Italy; 6 Fondazione IRCCS Casa Sollievo della Sofferenza, UO di Chirurgia Senologica, San Giovanni Rotondo, FG Italy; 7 Cancer Epigenetics and Biology Program (PEBC), Bellvitge Biomedical Biomedical Research Institute (IDIBELL), Barcelona, Catalonia Spain; 8 Centro de Investigación Biomédica en Red Cáncer (CIBERONC), Madrid, Spain; 9 Josep Carreras Leukaemia Research Institute, Badalona, Barcelona, Catalonia Spain; 10 Physiological Sciences Department, School of Medicine and Health Sciences, University of Barcelona, Barcelona, Catalonia Spain; 11 Institució Catalana de Recerca i Estudis Avançats (ICREA), Barcelona, Catalonia Spain

**Keywords:** Breast cancer, miRNAs, Predictive markers

## Abstract

MicroRNA-210-3p is the most prominent hypoxia regulated microRNA, and it has been found significantly overexpressed in different human cancers. We performed the expression analysis of miR-210-3p in a retrospective cohort of breast cancer patients with a median follow-up of 76 months (n = 283). An association between higher levels of miR-210-3p and risk of disease progression (HR: 2.13, 95%CI: 1.33-3.39, P = 0.002) was found in the subgroup of patients treated with Epirubicin and Cyclophosphamide followed by Docetaxel. Moreover, a cut off value of 20.966 established by ROC curve analyses allowed to discriminate patients who developed distant metastases with an accuracy of 85% at 3- (AUC: 0.870, 95%CI: 0.690-1.000) and 83% at 5-years follow up (AUC: 0.832, 95%CI: 0.656–1.000). Whereas the accuracy in discriminating patients who died for the disease was of 79.6% at both 5- (AUC: 0.804, 95%CI: 0.517–1.000) and 10-years (AUC: 0.804. 95%CI: 0.517–1.000) follow-up. *In silico* analysis of miR-210-3p and Docetaxel targets provided evidence for a putative molecular cross-talk involving microtubule regulation, drug efflux metabolism and oxidative stress response. Overall, our data point to the miR-210-3p involvement in the response to therapeutic regimens including Docetaxel in sequential therapy with anthracyclines, suggesting it may represent a predictive biomarker in breast cancer patients.

## Introduction

Breast cancer (BC) is the most frequent malignancy that develops in women worldwide and accounts for the highest rates of cancer-associated death. Indeed, despite the reported decline of breast cancer mortality in the last 15 years^1^, as a result of screening programs, better education, and implementation of more effective adjuvant treatments, about 25–40% of breast cancer patients develops metastases and eventually dies from the disease^2^. These data indicate that currently available prognostic and predictive methods are inadequate to determine precisely the aggressiveness of the disease and the likelihood of response to a certain treatment in individual patients.

microRNAs (miRNAs, miRs) are short (18–25 nucleotides), highly conserved non-coding RNAs that fine-tune gene expression by operating translation inhibition or degradation of protein coding mRNAs via imperfect base-pairing to complementary sites on target mRNAs. Soon after their discovery, microRNAs have been attracting much attention due to their frequent dysregulation in human cancers and, more importantly, their association with the clinicopathological parameters, the latter supporting their emergence as a new class of molecular biomarkers. In this regard, several miRNA profiles of breast cancer have been established by microarray or next-generation sequencing (NGS), and the resulting miRNA signatures have been correlated to the typical clinicopathological and prognostic characteristics including tumor size, lymph-node invasion, receptors status, and resistance to chemotherapy^3–6^.

Among these studies, a number of experimental evidences support the clinical relevance for miR-210. miR-210 is the most prominent hypoxia regulated microRNAs and a direct target of the hypoxia inducible factor 1 alpha (HIF-1α). Of the two mature forms, namely miR-210-3p and miR-210-5p, miR-210-3p appears to be the guide strand functionally involved in a variety of biological processes such as cell cycle, cell survival, stem cell differentiation, angiogenesis, DNA damage repair, mitochondrial metabolism, and immune response^7–9^. Moreover, as hypoxia is an important feature of solid tumors, the role of miR-210-3p has been extensively studied in the context of cancer progression, where it was shown that miR-210-3p is overexpressed in several malignancies including breast, glioblastoma, lung, pancreatic, or head and neck cancer^10–15^.

In breast cancer, the overexpression of miR-210-3p was initially correlated with a poor prognosis, being associated with invasiveness and shorter time to develop distant metastases^5,6,10,16–18^. In particular, miR-210-3p was shown to be up-regulated in triple-negative breast cancer (TNBC) compared to estrogen positive tumors^5,18^, showing relevance even in this group where high levels of miR-210 were found correlating with a higher risk of recurrence under tamoxifen treatment^19^. Interestingly, recent findings by Bar I. *et al*.^20^ demonstrated miR-210 tends to localize and be expressed both in epithelial cancer cells and in the tumor microenvironment (TME) of TNBC samples, particularly in inflammatory cells where it is likely to be regulated by a mechanism independent of HIF-1α^20^. In spite of these data, some discrepancies about miR-210-3p significance from a clinical standpoint do exist. For instance, by performing the expression profile of nine miRNAs in TNBC, Radojicic *et al*.^21^ recently reported that there was not a significant association between high expression levels of miR-210-3p and poor patient disease free survival and overall survival. Similar findings were reported by Makou A. *et al*.^22^ who found a significant association between miR-210-3p overexpression and shorter OS only in the ER-positive/HER2- negative breast cancer subtype.

These discrepancies spurred us to perform a miR-210-3p expression analysis in our large cohort of breast cancer patients with a long-term follow-up in the attempt to clarify the correlation of miR-210-3p with clinical parameters and help understand if miR-210-3p holds promise as biomarker in breast cancer.

## Results

### miR-210-3p is overexpressed in tumour samples as compared with normal breast tissues from reductive mammoplasty

The expression of miR-210-3p (hsa-miR-210-3p/RNU48x1000) was evaluated in 283 breast cancers (see Supplemental Table 1 for clinic-pathological data) and in the normal breast tissues obtained from 13 reductive mammoplasties (i.e. glandular tissue, NBTs). It emerged that miR-210-3p was significantly up regulated in tumours (Median 10.18; IQR 4.87-19.41) as compared with NBTs (Median 4.64; IQR 2.71–8.21) (P = 0.021) (data not shown).

### miR-210-3p up-regulation is more frequent in triple negative breast cancer

Then, we investigated the association between miR-210-3p expression and tumour clinicopathological characteristics. Higher levels of miR-210-3p expression were found in ER negative breast cancers (median 12.9, IQR 5.14–32.95) as compared with ER positive tumours (median 9.62; IQR 4.86–18.09) (P = 0.038).

When we compared cancer cases classified on the basis of surrogate molecular classification (Luminal A, Luminal B, *HER2*-amplified and Triple Negative), the Triple Negative subgroup showed the highest level of miR-210 expression (median 18.31 IQR 7.2–38.61), followed by Luminal B tumours (median 10.57 IQR 5.01–19.41), whereas Luminal A (median 8.6; IQR 4.2–15.09), and *HER2*-amplified tumours (median 8.57 IQR 4.66–24.69) showed the lowest expression levels (P = 0.0137). In detail, when pairwise comparisons were performed, we found a statistically significant difference between Luminal A vs. Triple Negative subgroup (Bonferroni-adjusted P = 0.0133) and also between Luminal B vs. Triple Negative (Bonferroni-adjusted P = 0.0181), respectively (Supplemental Table 2).

### Evaluation of miR-210–3p prognostic value in breast cancer cases

The association of miR-210-3p expression with time-to-event outcomes was evaluated in the group of patients without metastases at diagnosis (M0). Overall Survival (OS) and Progression Free Survival (PFS) data were available for 265 patients (Supplemental Tables S2, S3), whereas Metastases Free Survival (MFS) data were available for 263 patients (Supplemental Table S4). Tumour dimension, stage, lymph node status, hormone receptor status, *HER2-*amplification, and surrogate molecular classification were associated with PFS, MFS and OS. Instead, Ki67 overexpression was associated with PFS and MFS only. No statistically significant associations were found between miR-210-3p and outcomes in univariable analyses as well as in a multivariable model including clinical biomarkers indicated in the 8^th^ edition of the American Joint CC Breast Cancer prognostic classification^23^, which includes Pathological Stage (T = Tumour dimension, N = Lymph node status), Grade, HER2, ER and PgR status (Supplemental Table S5).

Next, we evaluated the association of miR-210-3p and patient’s outcome in the subgroup identified by adjuvant treatment (Supplemental Table S6). As shown in Table 1, miR-210-3p expression was associated in both univariable and multivariable analyses with an increased risk of disease progression and metastases development in the patient’s subgroup treated with the Epirubicin-Cyclofosfamide and Docetaxel (EC + D) scheme. No significant association was found within the Fluorouracil (5FU), Epirubicin and Cyclophosphamide (FEC) and Epirubicin-Cyclofosfamide (EC) schemes as well as no association was found in the group identified by endocrine and anti-HER2 therapies suggesting that miR-210-3p expression may affect the sensitivity of tumours treated with Docetaxel.

**Table 1:** Association between miR-210-3p expression and time-to-event outcomes according to adjuvant chemotherapy schemes.

Analysis	Time-to-event outcome	Chemotherapy	SD	N. total	N. events	HR	95%CI	p-value
Unadjusted	PFS	EC	111.596	43	10	1.33	0.96–1.85	0.085
		EC + D	16.622	54	11	2.13	1.33–3.39	0.002
		FEC	19.045	46	13	1.32	0.82–2.14	0.256
	OS	EC	111.596	43	4	Not est.	Not est.	Not est.
		EC + D	16.622	54	5	3.50	1.69–7.24	0.001
		FEC	19.045	46	10	1.21	0.72–2.03	0.474
	MFS	EC	111.596	42	8	0.69	0.15–3.28	0.644
		EC + D	16.622	53	10	2.35	1.51–3.67	<0.001
		FEC	19.045	46	15	1.12	0.70–1.80	0.627
Adjusted*	PFS	EC	111.596	39	10	1.34	0.94–1.91	0.105
		EC + D	16.622	52	10	2.14	1.26–3.63	0.005
		FEC	19.045	44	12	1.26	0.74–2.13	0.389
	OS	EC	111.596	39	4	Not est.	Not est.	Not est.
		EC + D	16.622	52	5	Not est.	Not est.	Not est.
		FEC	19.045	44	9	1.16	0.68–1.98	0.579
	MFS	EC	111.596	38	8	0.58	0.05–6.64	0.665
		EC + D	16.622	51	10	2.48	1.47–4.19	0.001
		FEC	19.045	44	13	1.01	0.64–1.61	0.957

Results are reported as hazard ratios (HRs), along with their 95% confidence interval (CI), which were expressed for each unitary increase in one standard deviation (SD) of miR-210-3p expression, as specifically detailed.

Abbreviations: Not est = HR not estimated because of failing in model’s convergence; PFS = Progression Free Survival; OS = Overall Survival; MFS = Metastases Free Survival; EC = Epirubicin + Cyclofosfamide; EC + D = Epirubicin + Cyclofosfamide + Docetaxel; FEC = 5-Fluoro-uracyl + Epirubicin + Cyclofosfamide).

*Adjusted HR estimated from Cox models which included prognostic factor according to 2018 AJCC 8^th^ classification Pathological Stage, Grade HER2/neu, Estrogen Receptor and Progesterone Receptor.

### High miR-210-3p expression can predict early metastases development and death in patients treated with epirubicin-cyclofosfamide and docetaxel (EC + D) regimen

**Figure 1:**
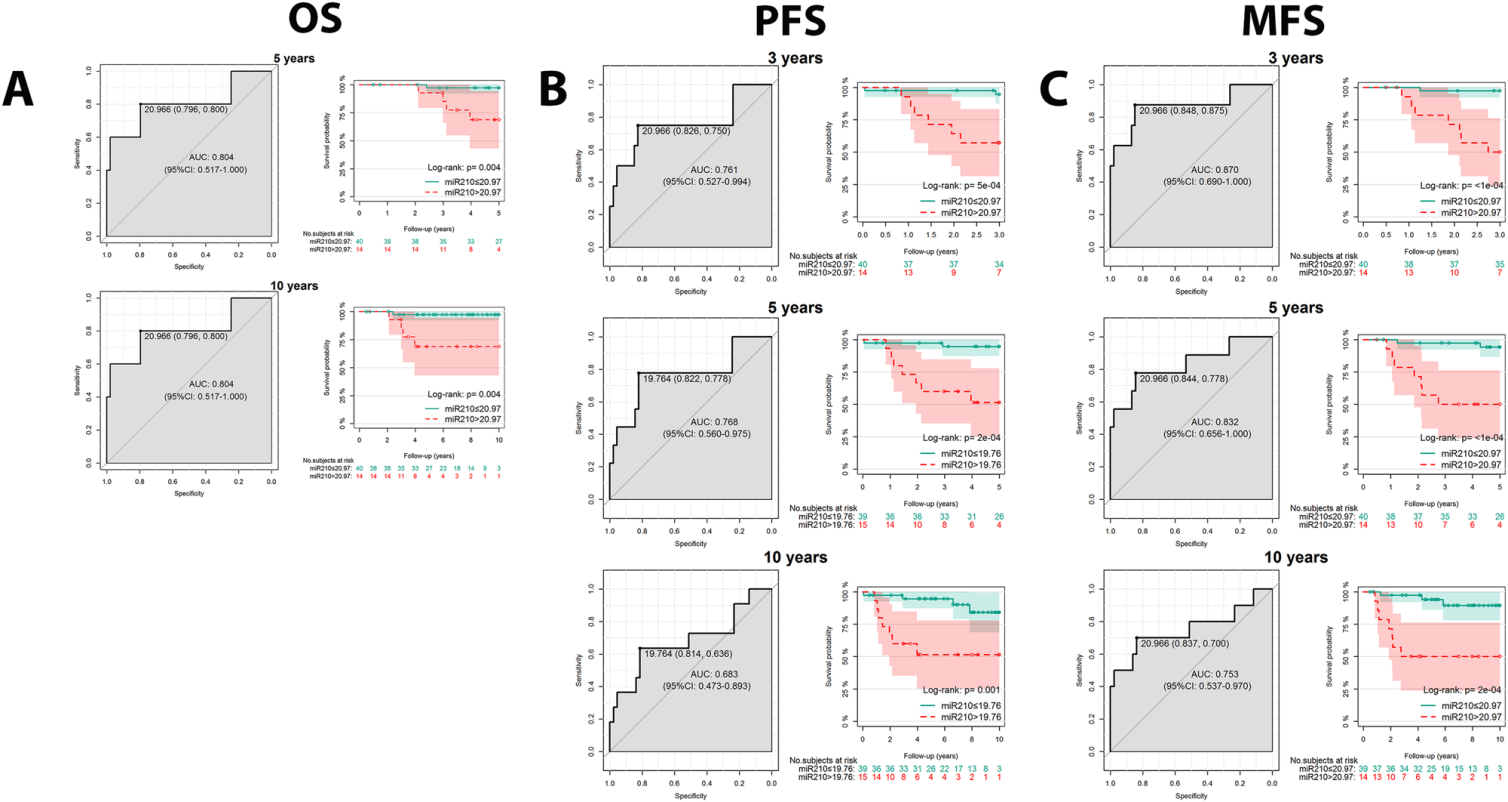
Prognostic ability of miR-210-3p expression in predicting survival outcomes in patients treated with Epirubicin-Ciclofosfamide and Docetaxel regimen. AUC curves and Kaplan Meier analysis are shown for (**A**) Overall Survival (OS), (**B**) Progression Free Survival (PFS), and (**C**) Distant Metastases Free Survival (MFS).

Based on the results obtained with survival analyses, we evaluated the discriminatory power of miR-210-3p expression analysis in identifying patients who experienced a time-to-event outcome. Again, the highest discriminatory power of miR-210-3p expression was achieved in the subgroup of patients treated with EC + D. Indeed, the prognostic ability of the miR-210-3p expression in discriminating patients who developed distant metastases reached significant AUC values of 0.87 (p < 0.001), 0.83 (p = 0.001) and 0.75 (p = 0.010) at 3, 5 and 10 years, respectively. On the basis of the ROC curve, the cut-off of 20.966 for miR-210 expression achieved the highest overall accuracies of 85.2% (sensitivity: 87.5%, specificity: 84.8%), 83.3% (sensitivity: 77.8%, specificity: 84.4%) and 81.1% (sensitivity: 70.0%, specificity: 83.7%) at 3, 5 and 10 years, respectively (Fig. 1C).

Moreover, the same cut off was also accurate in the discrimination of patients who died for the disease, with an overall accuracy of 79.6% (sensitivity 80%; specificity of 79.6%) and all miR-210-3p expression values achieved an AUC of 0.804 (p = 0.024) at both 5 and 10 years (Fig. 1A). Lastly, the prognostic ability of the miR-210-3p expression in identifying patients who developed a disease progression reached significant AUC values of 0.76 (p = 0.018) and 0.77 (p = 0.010) at 3 and 5 years, respectively. Again, the cut-off of 20.966 achieved the highest overall accuracy of 81.5% (sensitivity: 75.0%, specificity: 82.6%) at 3 years whereas the cut-off of 19.764 achieved the highest overall accuracy of 81.5% (sensitivity: 77.8%, specificity: 82.2%) at 5 years, respectively (Fig. 1B).

Finally, we evaluated the improvement in prognostic ability, in terms of survival C-statistics, passing from clinical to extended nested Cox models (i.e. clinical variables  +  miR-210-3p expression), among patients treated with EC + D. As shown in Table 2, although the nominal statistical significance was never reached for all tested models, slightly improvements were found in MFS at 3 and 5 years. Indeed, for MFS at 3 years, the clinical model achieved a survival C-statistic of 0.821 (95%CI: 0.707–0.935) and, with the further inclusion of miR-210 as covariate, such C-statistic increased to 0.891 (95%CI: 0.801–0.981) (improvement p-value = 0.102) whereas for MFS at 5 years, the clinical model achieved a survival C-statistic of 0.760 (95%CI: 0.602–0.917) and, with the further inclusion of miR-210-3p, such C-statistic increased to 0.868 (95%CI: 0.751–0.984) (improvement p-value = 0.077). These findings further support the hypothesis that miR-210-3p expression may predict therapy response in patients treated with docetaxel in sequential therapy with epyrubicin and cyclofosfamide.

**Table 2:** Improvement in prognostic ability from clinical Cox models (which included: pathological stage, grade, HER2/neu, estrogen receptor and progesterone receptor as clinical covariates) to extended Cox models, which included the covariates stated above and miR-210-3p expression, in patients treated with EC + D (N = 54).

				Survival C-statistic (95%CI*)		
Outcome#	Time (years)	N. total	N. events	Clinical model	Clinical + miR210 model	p-value°
PFS	3	52	7	0.769 (0.595-0.942)	0.860 (0.717–1.002)	0.250
	5	52	8	0.748 (0.595–0.902)	0.825 (0.656–0.993)	0.409
	10	52	10	0.741 (0.590–0.892)	0.757 (0.593–0.922)	0.805
MFS	3	52	8	0.821 (0.707–0.935)	0.891 (0.801–0.981)	0.102
	5	52	9	0.760 (0.602–0.917)	0.868 (0.751–0.984)	0.077
	10	51	10	0.764 (0.618–0.910)	0.821 (0.691–0.951)	0.318

Abbreviations: PFS = Progression Free Survival; MFS = Metastasis Free Survival. *95% confidence interval derived following perturbation-resampling methods;

°testing whether the prognostic ability achieved by the clinical model changed after the inclusion of miR-210-3p as prognostic covariate (i.e. two nested Cox models);

^#^Overall Survival outcome was not considered because HRs miR-210-3p expression could not be estimated in the multivariable Cox models.

### In silico prediction of miR-210-3p and docetaxel cross-talk

**Figure 2:**
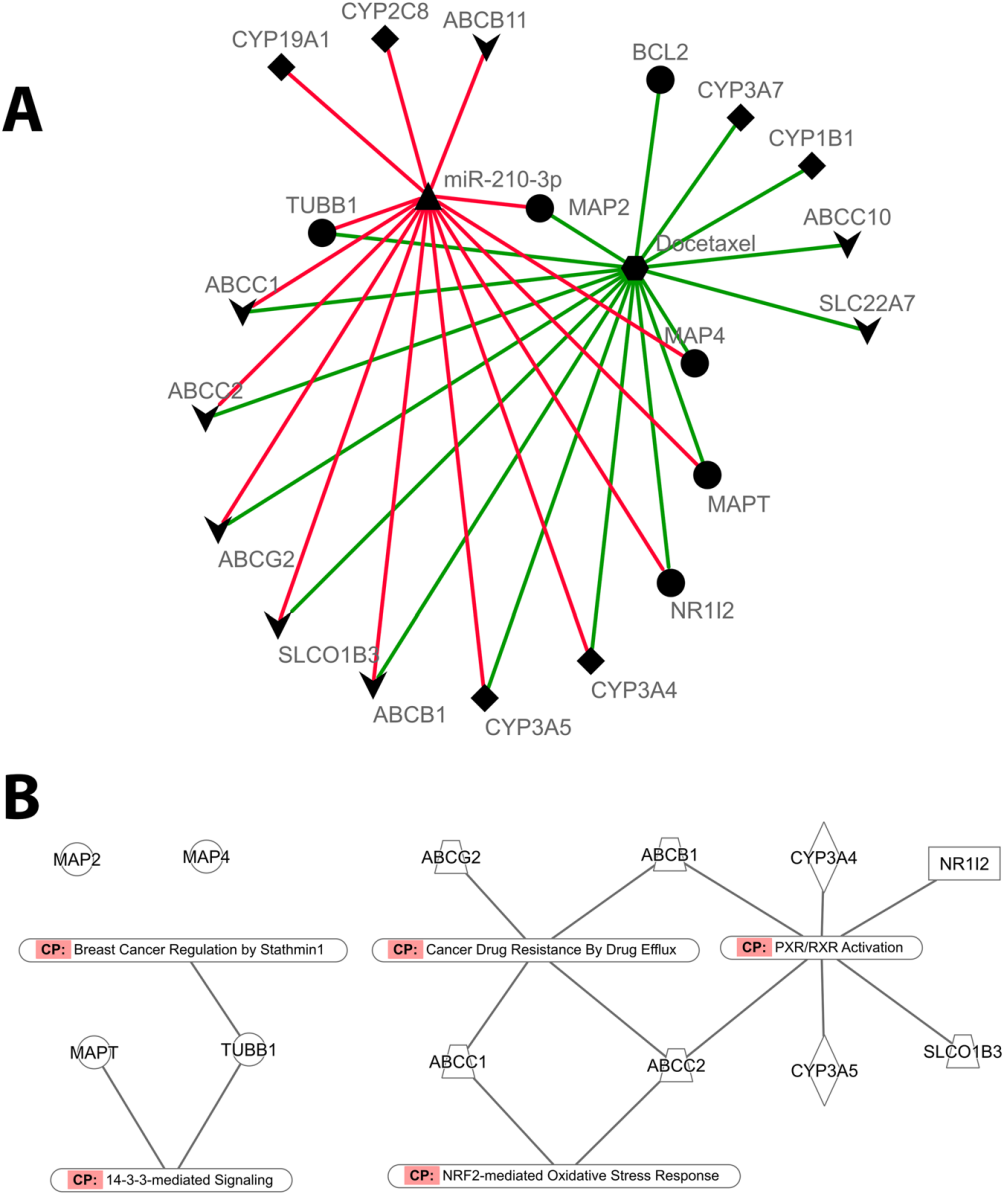
Identification of common molecules among miR-210-3p targets and Docetaxel interactors. (**A**) Molecular targets of miR-210-3p (red-linked) and Docetaxel (green). Circles are molecules which Docetaxel binds to, resulting in an alteration of the normal function of the bound molecule; Down-arrows are transporters; Rhombs are proteins which catalyze chemical reactions involving Docetaxel as substrate. (**B**) Known pathways which common molecules participate to.

In the attempt to provide biological relevance to our results, we interrogated the DrugBank and miRWalk databases to retrieve the molecular targets of Docetaxel and miR-210-3p, respectively (Fig. 2A). Twelve out of twenty targets were in common between the two, thereby suggesting being committed to a common molecular program. In particular, common genes resulted to be associated with the Cancer drug response by drug efflux and PXR/RXR activation pathways, as well as to Breast cancer regulation by Stathmin 1, 14-3-3-mediated signalling and NRF2-mediated oxidative stress response pathways (Fig. 2B).

## Discussion

To date, several studies have profiled the miR-210-3p expression in breast cancer patients and some of those have actually found significant correlations with the clinical outcome in support of the putative role that miR-210-3p might assume as biomarker in the management of BC patients^6,10,17,18,24^. In this context, due to the contradicting results we found in the literature, we took the effort to carry out the expression analysis of miR-210-3p in our cohort of 283 breast cancer patients followed by correlation analyses with the clinicopathological parameters to assess the capability to predict the clinical outcome of BC patients. As expected, miR-210-3p was significantly up regulated in tumours as compared with normal breast tissues. Moreover, consistently with previous findings, the Triple Negative BC molecular subgroup showed the highest levels of miR-210-3p expression, followed by Luminal B tumours, whereas Luminal A and *HER2*-amplified tumours showed the lowest expression levels.

Nevertheless, in agreement with a few reports^21,22,25^, the association of miR-210-3p with patient’ clinical outcome (i.e. OS, PFS and MFS) in the subgroup of patients without synchronous metastases did not reach the statistical significance in any molecular subgroup. This lack of significant associations with clinical endpoints in the total population suggested us to explore the possibility that miR-210-3p might be associated with outcomes within patient subgroups stratified according to treatment because of its documented involvement in hypoxia and, thereby, multidrug resistance^9,19,26–28^. In fact, when we performed the subgroup analyses evaluating the association of miR-210-3p with the response to adjuvant chemotherapy, we did not find any significant association with FEC and EC schedules but we did find that higher levels of miR-210-3p were associated with a 2-fold risk of disease progression under EC + D regimen. Moreover, when we evaluated the ability of miR-210-3p analysis in this adjuvant setting to identify those patients who progressed or died for the disease, we could establish a cut off value of 20.966 able to predict with a high sensitivity and specificity the development of distant metastases and overall survival at short-term and long-term follow up, respectively. The addition of miR-210-3p expression together with the clinical parameters indicated by the 8^th^ AJCC classification^23^ demonstrated a slight increase in survival C-statistic for the prediction of metastases free survival but without nominal statistical significance

Docetaxel is a second-generation taxane that, together with paclitaxel, belongs to the class of microtubule- stabilizing agents (MSAs) that bind to polymeric tubulin and prevent microtubule disassembly thus suppressing spindle microtubule dynamics, blocking mitosis and inducing apoptosis, and mitotic catastrophe following DNA damage. To uncover the molecular mechanisms underlying the role of miR-210-3p in resistance to Docetaxel we carried out a bioinformatic analysis that identified, among the targets of miR-210-3p and Docetaxel, twelve common molecules. (Fig. 2A) associated with microtubule regulation, drug efflux metabolism, and oxidative stress response (Fig. 2B). With regards to microtubule regulation, Docetaxel and miR-210-3p common targets participate to the 14-3-3 signaling that is involved in tubulin assembly^29^, and affects the function of Stathmin 1 (STNM1), a protein that destabilizes microtubule polymers of α-tubulin and β-tubulin subunits^30^. Interestingly, several studies suggest that the polymerization state of microtubules can affect the binding of antimicrotubule drugs. Indeed, increased microtubule polymerization augmented the binding of paclitaxel to microtubule^31,32^. Accordingly, decreased microtubule polymerization associated with overexpression of STNM1 decreased the binding of paclitaxel to breast cancer cells^33^.

It is well established that drug metabolism pathways play a pivotal role in multidrug resistance and our bioinformatic analysis also identified the drug efflux pathway, the PXR/RXR activation signaling and the NRF2-mediated Oxidative Stress Response as putatively involved in the interaction between miR-210-3p and Docetaxel targets. In particular, miR-210-3p expression is regulated at transcriptional level by the hypoxia inducible factor 1 alpha (HIF-1α) which directly binds to the hypoxia-responsive elements (HREs) at the *MIR210* gene promoter^34^. Interestingly, a recent study found anti-oxidant response elements (ARE) in the HIF-1α promoter, supporting the hypothesis that NRF2 activation sustains cell survival during hypoxia and hypoxia/reoxygenation^35^. Taken together, these data likely support a NRF2- HIF-1α-miR-210-3p axis as a further mechanism by which miR-210-3p might promote taxane resistance in cancer cells.

Although the analysis of treatment subgroups suffers from small sample size and, thus, low statistical power, to the best of our knowledge, this is the first study that brings into light a putative relationship between miR-210-3p and cancer responsiveness to taxanes in breast cancer and, hence, suggests the possibility to early predict the effectiveness of taxane-including schedules in patients undergoing adjuvant chemotherapy. The lack of previous evidences might be explained by taking into account that the subclassification and clinicopathological information of retrospective BC cohorts, with a long-term follow up, might be biased by the regular update of the clinical practice guidelines as well as the implementation of new therapeutic options, such as the adjuvant chemotherapy based on anthracyclines (doxorubicin, daunomycin, and epirubicin) and taxanes (paclitaxel and docetaxel). In addition to this, the different, often limited, sample size of analyzed cohorts, variations in technical and analytical procedures might be all responsible of the inconsistencies among the reports and highlight the need for standardization to make miRNA-based biomarkers of clinical utility.

## Conclusions

Overall, our data support the association of miR-210-3p with docetaxel and, therefore, the hypothesis that detecting the levels of miR-210-3p expression in the primary tumour may predict therapy response in patients treated with docetaxel in sequential therapy with anthracyclines. According to the ingenuity pathway analysis, hsa-miR-210-3p may affect pathways involved in both docetaxel metabolisms as well as in its function in microtubule stabilization. Thus, the increased expression of miR-210-3p may indicate a taxane-resistant prone cancer cell state.

## Materials and Methods

### Design, setting and eligibility criteria

This study, aimed at evaluating the expression profile of hsa-miR-210-3p in breast cancer, was carried out according to the REporting guidelines of tumor MARKer Studies (REMARK)^36^. A written research plan had been previously reviewed and approved by the Institutional Ethics Committee at IRCCS “Casa Sollievo della Sofferenza” (Prot N 140/CE). All the expression analyses were performed in snap-frozen fresh tissue specimens from a prospectively collected cohort of 283 breast cancer patients, characterized by a median follow-up of 75.6 (IQR 41.6–109.6) months. The eligible participants were females, aged more than 18 years, subjected to surgery at the Breast-Unit, IRCCS “Casa Sollievo della Sofferenza”, in the period between May 2005 and December 2014. Due to legal reasons, only one tumor specimen (approximately 50–100 mg of frozen tissue in weight) could be collected from each patient if the tumour was at least 1.0 cm in diameter. All patients provided prior written informed consent and all the procedures were performed according to International (Helsinki Declaration 7th rev, 2013, EU Directive 2004/23/EC) and Italian (D. Lgs. 30/06/2003, n. 196) regulations for research on human subjects.

#### Patients and treatment

The clinicopathological information of our patient cohort was collected from medical records and is presented in Supplemental Table S1. The median age of the study population is 59 years (range, 29 to 82), median tumor size is 2.5 cm (range, 0.5 to 11.0). Synchronous metastases were present in 15 cases whereas, among non-metastatic patients (N = 268), 55 experienced disease progression (Incidence Rate, IR 3.555 events per 100 PY) and 29 of them (IR 1.729 events per 100 PY) died for the disease.

Data were presented in accordance with recommendations for tumour biomarker prognostic studies^34^: In particular, tumors were considered estrogen receptor- (ER-) or progesterone receptor- (PgR-) positive when ≥1% of immunoreactive cells was detected^37^. HER2 status assessment was carried out according to standard recommendations^38^. Of the 283 patients, 106 (37%) were lymph node negative and 177 (63%) lymph node positive.

Due to the lack of reimbursement, multigene tests are currently not readily available for all patients in many countries including Italy. Consequently, the use of immunohistochemistry (IHC)-based biomarkers, such as Ki67, has been proposed in order to identify patients with low-risk outcome^39^. However, the 2015 St. Gallen panel proposed that Ki67 scores should be interpreted in light of local laboratory values, and recommended to use the median expression of each lab to define high and low values^40,41^. Thus, we used the median value in the study population, as cut off to distinguish low and high Ki67 expressing cases. According to this, Luminal A tumours were defined as HER2-negative, ER-positive, PgR positive with a low Ki67 assessment (<30%) and Luminal B-like tumours were defined as HER2-, ER-positive with a high Ki67 expression (≥30%). Overall, 103 cases (38%) were Luminal A, 94 cases (35%) were Luminal B; 33 *HER2*-amplified cases (12%), and 38 Triple Negative cases (14%). The remaining 15 cases were not classified because HER2 and/or Ki67 status was unknown. The patients of our cohort received adequate breast conserving surgery or total mastectomy and sentinel node biopsy or complete axillary dissection. Afterwards, the management of patients was performed as stated by to the following guidelines: AIOM (Associazione Italiana Oncologia Medica), St Gallen, NCCN and ASCO. Progression was defined as evidence of loco-regional (recurrence) and/or distant disease over 4 months from diagnosis and after curative-intent surgical treatment.

### RNA isolation and RT-qPCR for miRNA detection

The RNAs extracted from 500 ng of fresh frozen tissues were selected on the basis of RIN (RNA Integrity Number) calculation as previously described^42^. To assess miR-210-3p expression levels in our cohort of breast cancers we applied a relative quantification method (RT-qPCR) with standard curve (ref miR-9). Real-time PCR reactions were performed on ABI PRISM 7900HT Sequence Detection System (Thermo Fis Sc). For both miR-210-3p (ID 000512, Thermo Fisher Sc) and RNU48 endogenous control (ID 001006, Thermo Fisher Sc), standard curves were constructed by plotting the threshold cycle (Ct) values against logarithm10 of the copy number and fitting by linear least square regression. The level of miR-210-3p expression in each sample was determined following the method described previously^42^.

### Statistical analysis

Patients’ clinicopathological characteristics were reported as median along with interquartile range (IQR, i.e. first-third quartiles) for continuous variables, and frequencies for categorical variables. The two-sample t-test (or ANOVA model as appropriate) was used to evaluate differences in miR-210-3p expression among patient groups. In case of ANOVA model, pairwise comparisons among groups were performed and p-values were adjusted following the Bonferroni correction.

Normal distribution assumption was evaluated by Q-Q plots and Shapiro-Wilks test. These analyses demonstrated a log-normal distribution for miR-210-3p. Thus, all statistical analyses involving miR-210-3p expression were performed with their log transformed values.

Univariable and multivariable proportional hazards Cox regression models were used for time-to-event analyses, and risks were reported as Hazard Ratios (HR) and their 95% Confidence Interval (95%CI). miR-210-3p expression levels were considered both as continuous variables (i.e. HRs were expressed for each unitary increase in one standard deviation of expression levels) and as categorized variables, with respect to its median.

The time between the enrollment date and cancer related death was defined Overall Survival (OS), whereas the time-intervals between the enrollment date and tumour progression or metastases development were defined Progression Free Survival (PFS) and Metastasis Free Survival (MFS), respectively. Furthermore, the rates of mortality and disease progression were reported as number of events per 100 person-years.

The prognostic ability of miR-210-3p expression levels in discriminating patients who experienced a time-to-event outcome was assessed by the Area Under the Receiver Operating Characteristic (ROC) curves (AUC), censoring the follow-up time at different prediction time horizons (i.e. 3, 5 and 10 years), along with their 95% confidence intervals (95%CI), following DeLong method^43^. Moreover, the modified C-statistics for censored survival (i.e. “survival C-statistics”) data were estimated following Uno’s approach^44^ along with their 95% CI (confidence interval estimated using the perturbation-resampling method). The optimal cut-off which jointly maximizes both sensitivity and specificity in the ROC space was detected and plotted along with the ROC curve. Patients were therefore classified into two groups: those with the miR-210-3p expression lower the detected cut-off and those with the miR-210-3p expression greater than or equal to the detected cut-off. Kaplan-Meier curves were also plotted with respect to these two patients’ groups and log-rank test was performed. Furthermore, in the subgroup of patients treated with Docetaxel, improvement in prognostic ability achieved from two nested proportional hazards Cox models, was assessed following the method proposed by Uno’s^44^, concerning the inference for the difference in survival C-statistics between two competing risk prediction models. In detail, the first Cox model included the following clinical covariates: staging, grading, HER2/neu, estrogen receptor and progesterone receptor as clinical covariates (also used to perform multivariable Cox models) whereas the second (nested) one further included the miR-210-3p expression. The SAS Release 9.4 (SAS Institute, Cary, NC, USA) software was used to perform all statistical analyses, whereas plots were generated by using Comprehensive R Archive Network (CRAN) version 3.5.1 (packages: pROC, *survC1*, *survival*). A p-value below 0.05 was considered for statistical significance.

### Bioinformatic analyses

Molecular targets of miR-210-3p were retrieved from miRBase (version 3.0, accessed on October 2018). Among all targets, only experimentally verified targets were considered. DrugBank (version 5.1.1, accessed on October 2018) was queried in search of the molecular targets of Docetaxel. We divided the resulting targets into (i) proteins, macromolecules, nucleic acids, or small molecules to which Docetaxel binds, resulting in an alteration of the normal function of the bound molecule; (ii) enzymes that involve Docetaxel in their catalyzing functions; (iii) transporters that shuttle ions, small molecules or macromolecules across membranes, into cells or out of cells.

Common targets between miR-210-3p and Docetaxel were submitted to Ingenuity Pathway Analysis software and annotated with the molecular pathways that were involved in.

### Ethics approval and consent to participate

The experimental protocol was approved by the Ethical Committee of the Fondazione IRCCS “Casa Sollievo della Sofferenza” (Prot N140/CE). Prior written informed consent was obtained from all patients in accordance with the experimental protocol approved by the Ethical Committee.

## Supplementary information

Supplemental Tables

## Data Availability

The datasets analysed during the current study are available from the corresponding author on reasonable request.

## References

[1] Bray F (2018). Global Cancer Statistics 2018: GLOBOCAN estimates of incidence and mortality worldwide for 36 cancers in 185 countries. CA Cancer J. Clin..

[2] Harris LN (2016). Use of biomarkers to guide decisions on adjuvant systemic therapy for women with early-stage invasive breast cancer: American Society of Clinical Oncology clinical practice guideline. J. Clin. Oncol..

[3] Iorio MV (2005). MicroRNA gene expression deregulation in human breast cancer. Cancer Res..

[4] Blenkiron C (2007). MicroRNA expression profiling of human breast cancer identifies new markers of tumor subtype. Genome Biol..

[5] Foekens JA (2008). Four miRNAs associated with aggressiveness of lymph node-negative, estrogen receptor-positive human breast cancer. Proc Natl Acad Sci..

[6] Volinia S (2012). Breast cancer signatures for invasiveness and prognosis defined by deep sequencing of microRNA. Proc Natl Acad Sci..

[7] Qin Q (2014). Multiple functions of hypoxia-regulated miR-210 in cancer. J Exp. Clin. Cancer Res..

[8] Wang J (2014). Elevated expression of miR-210 predicts poor survival of cancer patients: a systematic review and meta-analysis. PLoS One.

[9] Bavelloni A (2017). MiRNA-210: a current overview. Anticancer Res..

[10] Camps C (2008). Hsa-miR-210 is induced by hypoxia and is an independent prognostic factor in breast cancer. Clin. Cancer Res..

[11] Puisségur MP (2011). miR-210 is overexpressed in late stages of lung cancer and mediates mitochondrial alterations associated with modulation of HIF-1 activity. Cell Death Differ..

[12] Malzkorn B (2010). Identification and functional characterization of microRNAs involved in the malignant progression of gliomas. Brain Pathol..

[13] Barbano R (2014). A miRNA signature for defining aggressive phenotype and prognosis in gliomas. PLoS One.

[14] Greither T (2010). Elevated expression of microRNAs 155, 203, 210 and 222 in pancreatic tumors is associated with poorer survival. Int. J. Cancer.

[15] Gee HE (2010). hsa-mir-210 is a marker of tumor hypoxia and a prognostic factor in head and neck cancer. Cancer.

[16] Buffa FM (2011). microRNA-Associated Progression Pathways and Potential Therapeutic Targets Identified by Integrated mRNA and microRNA Expression Profiling in Breast Cancer. Cancer Res..

[17] Rothe F (2011). Global microRNAs expression profiling identifies miR-210 associated with tumor proliferation, invasion and poor clinical outcome in breast cancer. PLoS One.

[18] Toyama T (2012). High expression of microRNA-210 is an independent factor indicating a poor prognosis in Japanese triple-negative breast cancer patients. Jpn. J. Clin. Oncol..

[19] Egeland NG (2015). The Role of MicroRNAs as Predictors of Response to Tamoxifen Treatment in Breast Cancer Patients. Int. J. Mol. Sci..

[20] Bar I (2017). The microRNA miR-210 is expressed by cancer cells but also by the tumor microenvironment in triple-negative breast cancer. J Histochem. Cytochem..

[21] Radojicic J (2011). MicroRNA expression analysis in triple-negative (ER, PR and Her2/neu) breast cancer. Cell Cycle.

[22] Markou A (2014). Prognostic Significance of Metastasis-Related MicroRNAs in Early Breast Cancer Patients with a Long Follow-up. Clinical Chemistry.

[23] Giuliano AE (2017). Breast Cancer-Major changes in the American Joint Committee on Cancer eighth edition cancer staging manual. CA Cancer J. Clin..

[24] Hong L (2012). High expression of miR-210 predicts poor survival in patients with breast cancer: a meta-analysis. Gene.

[25] Block MB (2018). Association of miR-548c-5p, miR-7-5p, miR-210-3p, miR-128-3p with recurrence in systemically untreated breast cancer. Oncotarget.

[26] Songchao L (2018). Downregulation of miR-210-3p encourages chemotherapy resistance of renal cell carcinoma via modulating ABCC1. *Cell &*. Bioscience.

[27] Grosso S (2013). MiR-210 promotes a hypoxic phenotype and increases radioresistance in human lung cancer cell lines. Cell Death & Disease.

[28] Forde JC (2012). Docetaxel maintains its cytotoxic activity under hypoxic conditions in prostate cancer cells. Urol. Oncol..

[29] Zhou Q (2010). 14-3-3 coordinates microtubules, Rac, and myosin II to control cell mechanics and cytokinesis. Curr. Biol..

[30] Andersen SS (2000). Spindle assembly and the art of regulating microtubule dynamics by MAPs and stathmin/Op18. Trends Cell Biol..

[31] Zhang CC (1998). The role of MAP-4 expression in the sensitivity to paclitaxel and resistance to Vinca alkaloids in p53 mutant cells. Oncogene.

[32] Zhang CC (1999). DNA damage increases sensitivity to Vinca alkaloids and decreases sensitivity to taxanes through p53-dependent repression of microtubule-associated protein 4. Cancer Res..

[33] Alli E (2002). Effect of stathmin on the sensitivity to antimicrotubule drugs in human breast cancer. Cancer Res..

[34] Dang K (2015). The role of hypoxia-induced miR-210 in cancer progression. Int. J. Mol. Sci..

[35] Lacher SE (2018). Identification of a functional antioxidant response element at the HIF1A locus. Redox Biol..

[36] McShane LM (2005). Statistics subcommittee of the NCI-EORTC working group on cancer diagnostics. Reporting recommendations for tumor marker prognostic studies. J. Clin. Oncol..

[37] Hammond ME (2010). American Society of Clinical Oncology/College of American Pathologists guideline recommendations for immunohistochemical testing of estrogen and progesterone receptors in breast cancer. J. Clin. Oncol..

[38] Wolff AC (2013). Recommendations for human epidermal growth factor receptor 2 testing in breast cancer: American Society of Clinical Oncology/College of American Pathologists clinical practice guideline update. J. Clin. Oncol..

[39] Goldhirsch A (2011). Strategies for subtypes–dealing with the diversity of breast cancer: highlights of the St. Gallen International Expert Consensus on the Primary Therapy of Early Breast Cancer. Ann. Oncol..

[40] Coates AS (2015). Tailoring therapies–improving the management of early breast cancer: St Gallen International Expert Consensus on the Primary Therapy of Early Breast Cancer. Ann. Oncol..

[41] Senkus E (2015). Primary breast cancer: ESMO Clinical Practice Guidelines for diagnosis, treatment and follow-up. Ann. Oncol..

[42] Barbano R (2017). Stepwise analysis of MIR9 loci identifies miR-9-5p to be involved in Oestrogen regulated pathways in breast cancer patients. Sci Rep..

[43] DeLong ER (1988). Comparing the areas under two or more correlated receiver operating characteristic curves: a nonparametric approach. Biometrics.

[44] Uno H (2011). On the C-statistics for evaluating overall adequacy of risk prediction procedures with censored survival data. Statistics in medicine..

